# PMMA/pPFPA membrane with low content of modified TiO_2_ nanoparticles for effective retention of pharmaceuticals from water

**DOI:** 10.1038/s41598-026-45387-3

**Published:** 2026-03-26

**Authors:** Mariia Pasichnyk, Christian Schmitt, Martina Plank, Kristin Kerst, Wei Wang, Muhannad Al Aiti, Gianaurelio Cuniberti, André Lerch, Patrick Théato

**Affiliations:** 1 Chair of Process Engineering in Hydro Systems, TUD Dresden University of Technology, 01069 Dresden, Germany; 2 Institute for Technical Chemistry and Polymer Chemistry (ITCP), Karlsruhe Institute of Technology (KIT), Engesserstraße 18, 76131 Karlsruhe, Germany; 3 Institute for Biological Interfaces III (IBG-3), Soft Matter Synthesis Laboratory, Karlsruhe Institute of Technology (KIT), Hermann-von-Helmholtz-Platz 1, 76344 Eggenstein-Leopoldshafen, Germany; 4 Chair of Material Science and Nanotechnology, TUD Dresden University of Technology, 01062 Dresden, Germany

**Keywords:** Chemistry, Environmental sciences, Materials science, Nanoscience and technology

## Abstract

In this work, we addressed the issue of pharmaceutical pollution in water by developing new polymer-based membranes with superior separation and photocatalytic properties. The membranes were prepared via the phase-inversion method using poly(methyl methacrylate) (PMMA) and poly(pentafluorophenyl acrylate) (pPFPA) as the main polymers. To enhance absorption capacity and activate photocatalytic properties, modified TiO₂ nanoparticles (TiO_2, mod_.) were introduced in low concentration. Two porous membranes were fabricated: M1, consisting of PMMA, pPFPA, and TiO_2, mod_. nanoparticles; and M2, containing the same components as M1 but additionally supplemented with high molecular weight poly(ethylene glycol) (PEG) and polyvinylpyrrolidone (PVP). The incorporation of high-molecular-weight PEG and PVP reduced void formation in the membrane structure, resulting in a denser morphology with smaller pores. The membrane morphology and surface properties were characterised using scanning electron microscopy (SEM), Fourier transform infrared spectroscopy (FTIR), zeta potential measurements, and captive bubble contact angle analysis. The average pore diameter decreased from 0.025 μm to 0.0037 μm (85% reduction), resulting in a more uniform pore-size distribution and enhanced membrane structural stability. The surface hydrophilicity, measured using the captive bubble method, improved from 20° to 15°, corresponding to a 25% decrease in contact angle, indicating enhanced hydrophilicity of the PMMA/pPFPA/TiO_2, mod_./PEG/PVP membrane. The membrane was tested against a pharmaceutical mixture of metoprolol (MPL), ibuprofen (IBU), and diclofenac (DCF) in dynamic (cross-flow) and static (photocatalysis, sorption) modes. The cross-flow membrane rejection of pharmaceuticals followed the order DCF < IBU < MPL and did not exceed 30%. Independent sorption experiments indicated retention rates of 50–70%, while photocatalytic degradation achieved complete removal (100%) within 2 h. These results clearly separate the contributions of rejection, sorption, and photocatalysis, highlighting the membrane’s potential for practical applications in water treatment.

**Supplementary Information:**

The online version contains supplementary material available at 10.1038/s41598-026-45387-3.

## Introduction

The presence of pharmaceuticals in drinking water and their potential impact on human health are often underestimated but remain a serious concern. As a result, drinking water is a growing and significant source of certain pharmaceuticals that are unintentionally consumed, compared to other pollutants. It is important to note that these pharmaceutical contaminants are currently detected in drinking water systems, posing measurable environmental and health-related risks^1^.

Many pharmaceuticals are on the watch list under the Water Framework Directive (2013/39/EU), raising questions about the regulation of pharmaceutical contaminants in drinking water. The European Parliament resolution of 17 September 2020 on a strategic approach to pharmaceuticals in the environment (2019/2816(RSP)) has highlighted that pharmaceutical products and residues are commonly found in water bodies and indicated that wastewater treatment plants (WTP) can reduce the presence of pharmaceuticals in drinking water, but they are not able to completely eliminate them^2^. Research suggests that adsorption and advanced oxidation processes (AOPs) have the potential to treat pharmaceutical pollutants^3^. However, the conventional activated sludge process (CAS) is not useful in removing pharmaceuticals^4^.

New solutions for removing pharmaceuticals from drinking water sources require the urgent utilisation of innovative approaches and a combination of diverse technologies. Membrane technology, tertiary biological processes, AOP^5^, photodegradation^6^ and adsorption^7^, have demonstrated their effectiveness, achieving a removal efficiency of up to 99% for targeted pharmaceuticals. Equally promising results have been reported for hybrid treatment approaches that integrate membrane processes with photodegradation or advanced oxidation processes for pharmaceutical removal^8^. Among these advanced strategies, membranes with photocatalytic functionality have attracted particular attention for their ability to combine separation and degradation in a single step. During photocatalysis on the membrane surface, hydroxyl radicals, powerful oxidants and reductants, are generated. These radicals degrade organic pollutants, such as pharmaceuticals, into simpler, smaller, and less harmful inorganic molecules without generating secondary waste. Reactive radicals can be produced by energy sources such as UV light^9^. Therefore, the efficiency of photocatalytic membranes strongly depends on the accessibility and distribution of the photocatalyst on or near the membrane surface.

As nanoparticles are incorporated into the membrane, their morphology is also important. Most previous studies have chosen titanium dioxide (TiO_2_) as a photocatalyst to prepare photocatalytic membranes due to its long electron-hole pair lifetime, stability across a wide pH range, strong catalytic activity, and well-known features as a cost-effective material^10^. Alternative photocatalysts, including tungsten oxide^11^, Cu-doped graphene oxide^12^ and metal–organic frameworks (MOFs) such as Zn-MOF^13^, have also been explored for photocatalytic pharmaceutical removal. However, these alternative materials often face practical challenges, such as lower stability, narrower working pH ranges, or more complex synthesis, leaving TiO_2_ as the most established option for photocatalytic membranes.

Polyvinylidene fluoride (PVDF)-based TiO_2_ nanocomposite membranes have been widely used for photocatalytic removal of pharmaceuticals from water due to PVDF’s excellent mechanical strength, chemical resistance, and processability. In addition to photocatalytic degradation, PVDF/TiO_2_ membranes^14^ have demonstrated measurable pharmaceutical rejection via size exclusion, adsorption, and electrostatic interactions. The incorporation of TiO_2_ often enhances membrane hydrophilicity, which can influence solute–membrane interactions and contribute to improved rejection performance. When creating such membranes, nanoparticles are embedded in the polymer matrix during membrane formation, partially covering the active sites and limiting their accessibility for surface-driven photocatalytic reactions. Furthermore, the hydrophilic hydroxyl groups on TiO_2_ nanoparticles reduce the compatibility between the inorganic nanoparticles and the polymer matrix, leading to increased nanoparticle agglomeration. Fischer et al. developed a method for in situ growth of TiO_2_ nanoparticles on wet PVDF membranes via hydrolysis^15^, enabling strongly attached, non-agglomerated anatase nanoparticles that improved photocatalytic degradation of pharmaceuticals while reducing membrane fouling. However, this synthesis involves multiple controlled steps, relies on precise hydrolysis and vapour-phase crystallisation conditions, and may be difficult to scale or transfer to ultrafiltration membranes. Additionally, the weak interaction between the matrix and the TiO_2_ nanoparticles increases the risk of their leakage into the environment. Long-term stability of TiO_2_ immobilisation within polymeric membranes has been previously demonstrated using polyacrylic acid (PAA) binding on polyethersulfone membranes, where sustained nanoparticle retention and stable membrane performance were observed during extended operation^16^. Such studies emphasise the importance of strong interfacial interactions for preventing nanoparticle leaching and ensuring long-term membrane reliability.

To ensure effective and long-lasting wastewater treatment, photocatalytic membranes with strong nanoparticle binding stability are required. In this context, chemical bonding offers a robust strategy to securely attach the TiO_2_ nanoparticles to the membrane surface, preventing shedding and extending the membrane’s lifespan in water^17^. (3-Aminopropyl)triethoxysilane (APTES) modified TiO_2_ nanoparticles characterised with great improvement in photocatalytic activity due to the presence of silicon and carbon. This effectively delays the anatase–rutile phase transformation, as shown in the works of Tobaldi et al.^18^, Okada et al.^19^, and Morawski et al.^20^, and potentially suppresses the agglomeration tendency of nanoparticles due to the repulsive interaction of the terminal amino groups. These primary amino groups can easily form covalent bonds with activated esters, which are widely used in material science^21^. Moreover, chemical bonding to stabilise TiO_2_ nanoparticles on the membrane surface is expected to enhance photocatalytic performance.

For membrane fabrication with APTES-modified TiO₂, the polymer matrix should contain a high density of reactive functional groups to form stable covalent bonds and ensure structural integrity; typical PVDF is chemically inert and lacks sufficient active sites, whereas poly(pentafluorophenyl acrylate) (pPFPA) is an excellent candidate for preparing various functional polymers^22^, nanofibers^23^ and biologically active compounds^24^.

However, membranes based on pPFPA have not been explored broadly. Rachelle M. Arnold et al. have shown that to reduce hydrolysis and anhydride formation during pPFPA functionalization, it could be covalently attached to silicon oxide to form a stable product^25^. The PFPA groups can react with primary amines of APTES-modified TiO_2_ nanoparticles, forming a chemical bond, thus preventing shedding and improving reusability. This covalent immobilisation strategy also provides a strong justification for the use of pPFPA, even though fluorinated polymers are generally considered critical for application in water filtration. A further point for consideration is whether pPFPA itself leaches from the membrane, which requires careful evaluation to ensure long-term stability and safety. Moreover, it was reported that fluoride groups can easily interact with different pharmaceuticals^26^, increasing the retention rates of membranes^27^, while immobilisation of TiO_2_ nanoparticles improves photocatalytic degradation of pharmaceuticals on the membrane surface^28^. A fundamental understanding of this should help in developing next-generation membranes for targeted solute removal.

Mixing pPFPA with different polymers in the presence of diamines can create a cross-linked structure^29^. Such a polymer network affects solute transport and may obscure the role of specific solute-polymer interactions. Poly(methyl methacrylate) (PMMA) is particularly interesting due to its good compatibility with pPFPA and other polymeric additives^24^. The blend membranes are characterised with enhanced hydrophilicity and water permeation fluxes compared to pure PMMA membranes. For example, incorporation of carboxyl functional groups can promote the adsorption of cationic pharmaceuticals^30^. Fluorinated polymers generally show low compatibility with other polymers; however, by controlling the blending ratio (e.g., 1:1), a predictable pore structure and crosslinked network can be formed^31^.

Moreover, to control membrane porosity, morphology, and surface properties, pore-forming agents can be added to the membrane composition. The leading among them remains PEG. However, it was discovered that the molecular weight of PEG plays a critical role in pore formation. Low-molecular-weight PEGs diffuse rapidly during coagulation, typically producing larger and more open pores^32^. In contrast, high-molecular-weight PEGs increase solution viscosity and minimise solvent–nonsolvent exchange, leading to smaller, more uniform pores and reduced macrovoids^33^. Similarly, higher-molecular-weight PVP can also suppress macrovoids, forming a denser and more homogeneous membrane structure. This approach offers the possibility of adjusting the pore morphology and membrane structure by selecting appropriate molecular weight additives.

In this study, we developed blend membranes of pPFPA and PMMA to combine the benefits of both polymers, including improved mechanical resistance, processability, and ionic conductivity. We further investigated the photocatalytic activity of pPFPA/PMMA membranes incorporating APTES-modified TiO_2_ nanoparticles. The covalent bonding of TiO_2_ to pPFPA enhances membrane stability and effectively prevents nanoparticle release from the surface. Most studies proposed adding nanoparticles to the casting solution in a relatively high concentration, i.e., above 1 wt%^34^. However, negative effects of high concentrations on the performance of the resulting membrane have also been observed^35^. This study focused on adding a low concentration of modified TiO_2_ nanoparticles (TiO_2, mod_) to the polymer composition to enhance the membranes’ absorption capacity and activate photocatalytic properties. Notably, only a low TiO_2_ content (0.5 wt%) was employed, ensuring that active sites were covalently bound, thereby minimising potential leaching and reducing harmful effects. High-molecular-weight PEG and PVP were added to form a more uniform pore structure. The hybrid system fills the hydrophobic macroporous structure with a crosslinked hydrophilic network (from PFPA and PEG). A small amount of PVP, due to its solubility in the precipitation bath and its lack of crosslinking ability, was included to maintain pore formation and ensure a homogeneous distribution of PEG and TiO₂, which supports high selectivity and retention without compromising flux. The study focused on the rejection of pharmaceuticals, including non-steroidal anti-inflammatory drugs such as diclofenac (DCF) and ibuprofen (IBU), as well as the cardiac medication metoprolol (MPL).

## Results and discussion

**Fig. 1:**
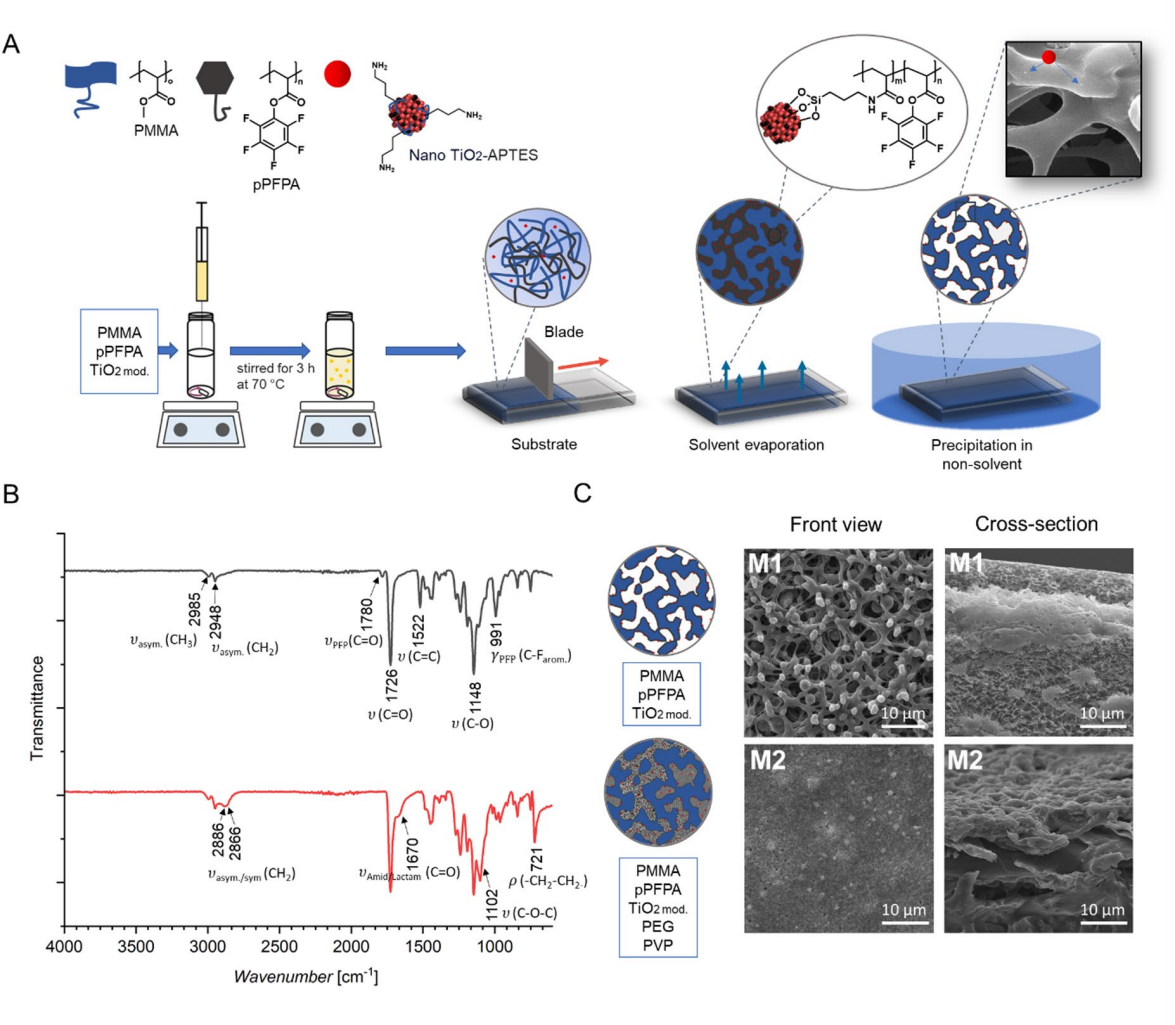
Surface and characteristics of the prepared membranes: (**A**) Schema of the membrane preparation including interactions between PMMA, pPFPA and modified TiO_2_ nanoparticles; (**B**) FTIR transmittance spectra of M1 (PMMA/pPFPA/TiO_2 mod_) and M2 (PMMA/pPFPA/TiO_2 mod_/PEG/PVP) membranes in the wavenumber range of 4000–350 cm⁻¹; (**C**) SEM images of M1 (PMMA/pPFPA/TiO_2 mod_) and M2 (PMMA/pPFPA/TiO_2 mod_/PEG/PVP) membranes – front view and cross-section.

Two composite membranes with a 1:1 ratio of PMMA to pPFPA were created using nonsolvent-induced phase separation and studied (Fig. 1A). M1 (PMMA/pPFPA/TiO₂ mod) was prepared from the hydrophobic polymer blend, and M2 (PMMA/pPFPA/TiO_2 mod_/PEG/PVP) is an improved version with optimised pore structure for the intended application.

The presence of APTES groups on the surface of TiO_2 mod_ nanoparticles enable covalent binding to pPFPA via amidation (Fig. 1A). The prepared membranes contain nanoparticles firmly held within the polymer matrix by covalent amide bonds, preventing their release into the environment during use. The low nanoparticle content, combined with APTES modification, ensures that all nanoparticles can react with the active sites of pPFPA^36^, thereby improving stability and performance without the drawbacks associated with higher loadings (Fig. 1A).

### Characteristics of the newly developed membrane

#### Fourier-transform infrared spectroscopy (FTIR)

Figure 1B shows the FTIR spectra of the produced membranes. The spectra in black (top), representing membrane M1, show the asymmetric stretching vibrations of methyl and methylene groups of PMMA appeared at 2985 and 2948 cm⁻¹, while the strong 1726 cm⁻¹ attributed to ester C = O stretching^37^. The peaks at 1148 and 991 cm⁻¹ corresponded to C–O and C–F vibrations originating from the pPFPA segments. The peaks at 1780 and 1522 cm⁻¹ can be assigned to the perfluorophenyl carbonyl and aromatic modes of the pPFPA. The existence of these peaks proves that the polymer blend was successfully prepared^38^. It is shown in direct comparison with the red spectra (bottom), which refer to membrane M2, and differs by adding the additives PEG and PVP to the composition of M1. Here, the pPFPA related peaks at 1780 and 1522 cm⁻¹ disappeared, and a new band at 1670 cm⁻¹, indicating the presence of amide or lactam, was assigned^19^. Moreover, an additional CH₂ stretching band at 2886 cm⁻¹, a strong band at 1102 cm⁻¹ (C–O–C) and a –CH₂–O rocking mode at 721 cm⁻¹ appeared which is typical for the PEG^39^. Additionally, the newly formed CH_2_-stretching band indicate an overlapping of two bands, which is combined with the band at 1670 cm⁻¹, a clear hint that PVP is also still integrated^40^. These changes confirm that the PEG and PVP additives were successfully integrated into the polymer composite, introducing ether and amide functionalities and modifying the carbonyl region compared to the membrane without additives. For membrane M2, it can be assumed that in solution the highly active pPFPA polymer forms covalent bonds with the hydroxyl end groups of PEG, forming a brush-to-network structure in which PVP is integrated due to the high coordination capability of the lactam free electron pair. Furthermore, this network allows TiO₂ particles to form covalent bonds with pPFPA and coordinative interactions with PEG and PVP. Due to polarity differences, phase separation occurs, resulting in a PMMA matrix with a hydrophilic pore structure composed of polyacrylic acid (PAA), cross-linked with PEG, coordinated with PVP and containing embedded modified TiO₂ nanoparticles. FTIR analysis confirmed the complete transformation of pPFPA. Some intermolecular associations in the interfacial and disordered regions of PMMA and pPFPA are broken upon PEG insertion between chains.

#### Scanning electron microscopy (SEM)

**Fig. 2:**
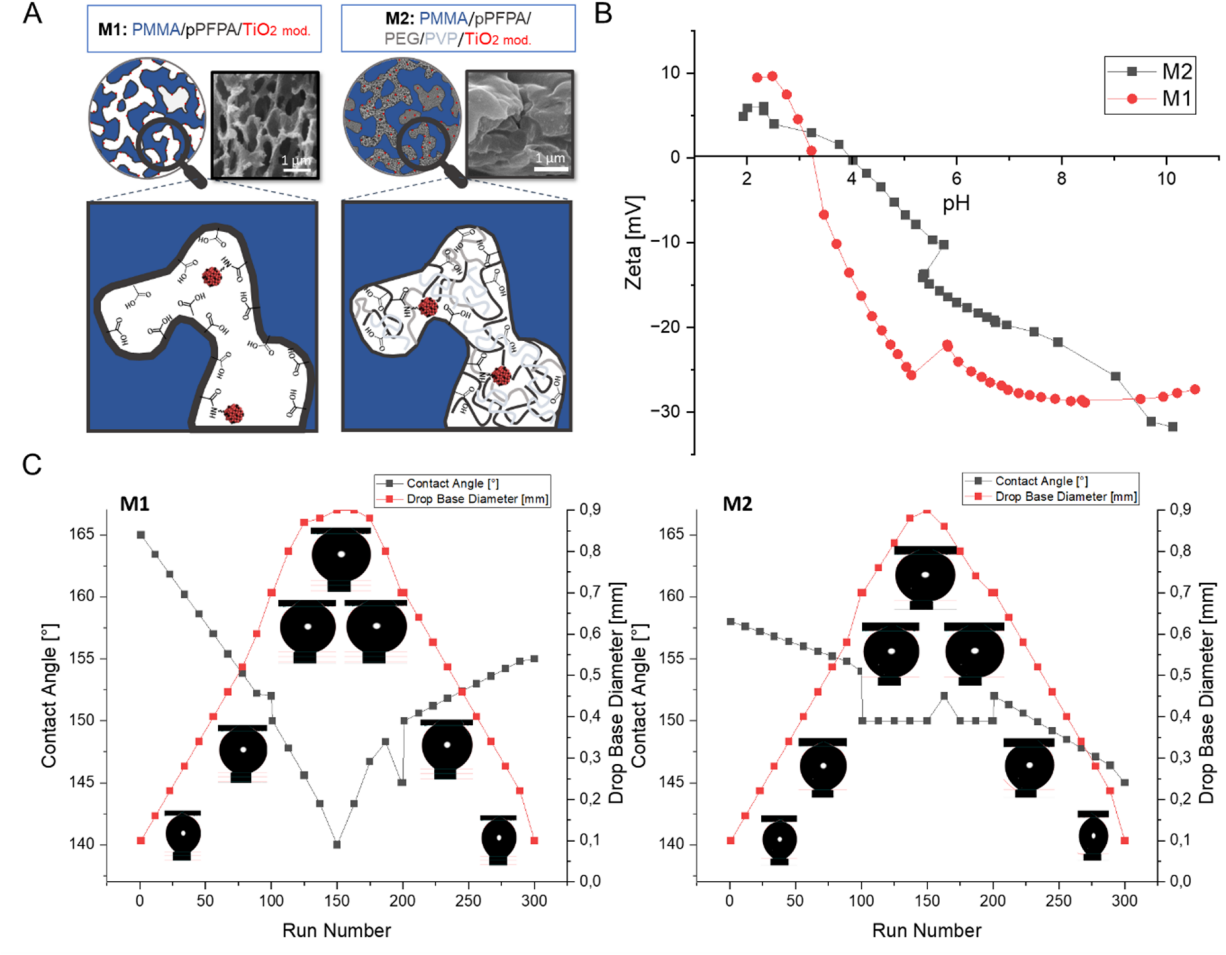
Surface and electrokinetic characteristics of the prepared membranes: (**A**) Schematic representation of the expected chemical composition of the pore structure with a close-up of the corresponding SEM cross-section; (**B**) Zeta potential of the surface of M1 (PMMA/pPFPA/TiO_2 mod_) and M2 (PMMA/pPFPA/TiO_2 mod_/PEG/PVP) membranes; (**C**) Contact angle values by the captive bubble method of M1 (PMMA/pPFPA/TiO_2 mod_) and M2 (PMMA/pPFPA/TiO_2 mod_/PEG/PVP) membranes dependent on drop volume (0.1–0.9 µL), time 100 s.

SEM images shown in Fig. 1C revealed that the TiO_2, mod_. nanoparticles were evenly distributed on the membrane’s surface, and no significant cluster formation was observed, indicating good nanoparticle dispersion. Membrane M1 (Fig. 1C) without additives showed a macroporous interconnected network with open pores with an average pore diameter of 2.0 ± 0.5 μm and a pore density of 0.025 pores/µm². The cross-section morphology of the membrane displayed a typical asymmetric structure with spongy voids with a thickness up to 40 μm (see Figs. 1C and 2A). The membrane with additives also showed on the surface a homogeneous pore distribution, even though the pores are smaller by nearly one order of magnitude, as can be directly seen by comparing both membranes in Fig. 1C. Image analysis revealed a lower pore density of 0.0037 pores/µm² and much smaller pores, with an average diameter of 0.30 ± 0.11 μm. The cross-section of M2, in contrast to M1, shows a dense and thin polymer layer of less than 10 μm.

The SEM analysis of the membrane cross-section revealed a dense structure with clustered domains, suggesting incompatibility between the polymer components. The different polarities of the components led to phase separation and resulted in compositional inhomogeneity, as expected.

Membrane M1 showed the typical asymmetric membrane structure generated by spinoidal demixing in non-solvent^41^. Here, it is assumed that only the interface with the voids bears carboxylic acid groups due to hydrolysis during the phase inversion process (Fig. 2A)^42^. The core phase of the pPFPA remained intact, as supported by the FTIR data (Fig. 1B). For membrane M2, a change in the polarity of pPFPA due to the esterification of PEG and pPFPA, resulted in different phase separation behaviour. Thus, a dense, clustered layer was formed, revealing a homogeneous pore structure. It is assumed that PMMA, as a hydrophobic polymer, forms the matrix, whose pores are filled with a network of pPFPA/PEG/PVP/TiO₂ particles (Fig. 2A). Since the pPFPA was now homogeneously distributed in the hydrophilic pores, penetrating water hydrolysed the remaining PFPA groups quantitatively, within the phase inversion, as confirmed by the FTIR data, where the IR bands indicating the pPFPA have disappeared (Fig. 1B). It is worth mentioning that the pore size of the substrate lies in the submicrometer range, and the M1 polymer blend composition was not enough to cover these submicrometer pores.

#### Surface charge of the membrane

The interaction between the membrane surface and feed water compounds was influenced by the membrane’s surface charges. The surface charge of the membrane was determined by measuring the zeta potential, as illustrated in Fig. 2B. The membrane’s zeta potential was positive for pH levels below 4 and became negative for pH levels above 4. For the M1 membrane, the isoelectric point (IEP) was at 3.15, while for the M2 membrane, it was at 3.98 (Fig. 2B). The point zero charges of the M2 membrane, with additives PEG and PVP, shifted slightly towards more positive values; however, the membrane remained negatively charged. In the pH range of 2–10, the zeta potential values ranged from 10 to -40 mV for M1 and 6 to -31 mV for M2. As the pH changes from acidic to alkaline, the surface charge of the membrane became more negative. At pH 6, the charge of the membranes was − 25 mV and − 17.7 mV, respectively, indicating the presence of more negatively charged phases on the surface of the membrane without PEG and PVP (Fig. 2B). These findings are consistent with FTIR results (Fig. 2), which identified a negatively charged carbonyl group at the peak of 1726 cm^− 1^. Surface charge is used to predict the potential of the membrane to attract pharmaceutical fouling. Charged surfaces created repulsive forces, thereby reducing the deposition of solutes on the charged membrane surface.

#### Contact angle measurements by the captive bubble method

**Fig. 3:**
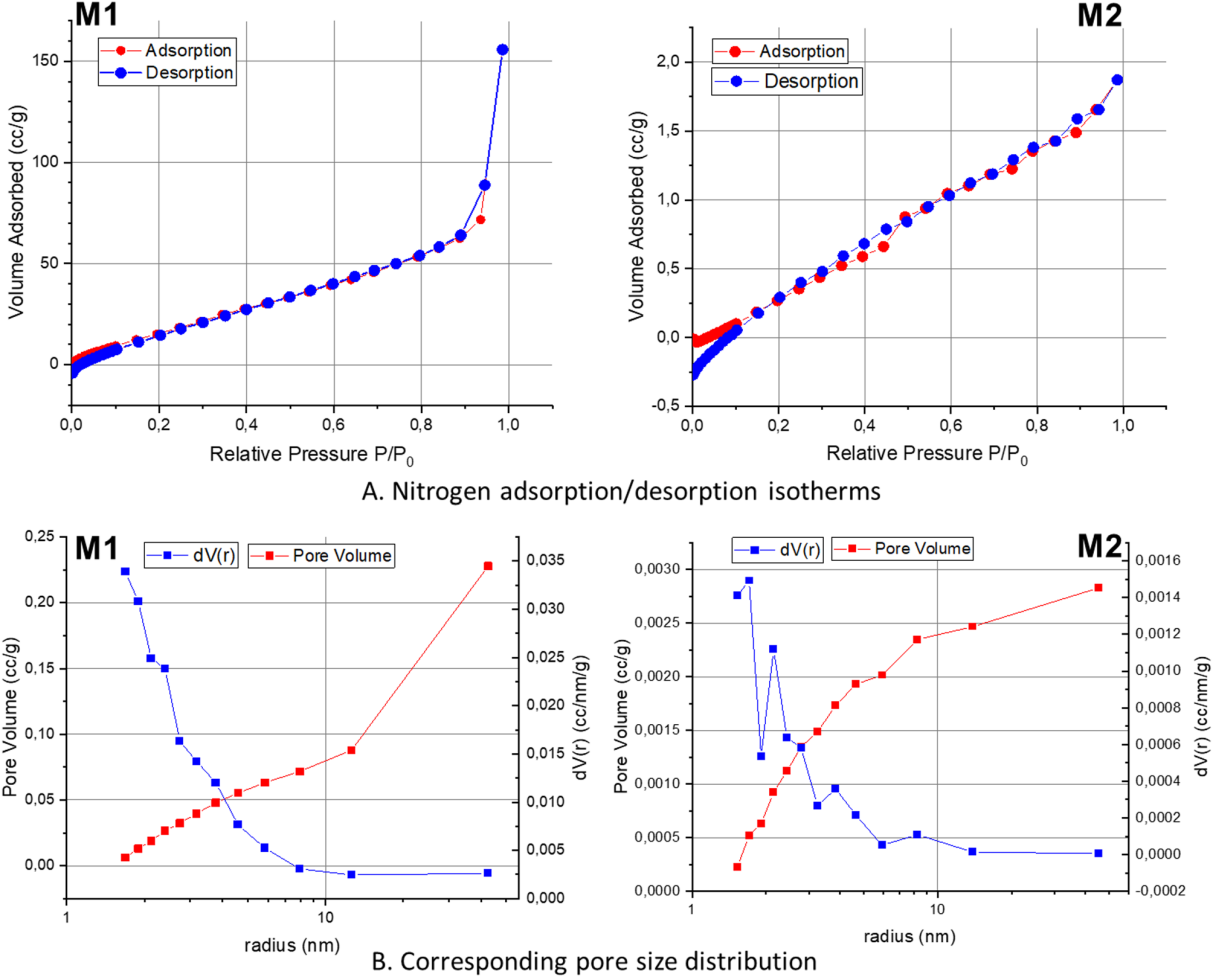
Nitrogen adsorption–desorption isotherms and BET analysis showing specific surface area and pore size distribution for M1 (PMMA/pPFPA/TiO_2 mod_) and M2 (PMMA/pPFPA/TiO_2 mod_/PEG/PVP) membranes.

Figure 2C shows the results of the contact angle measurements by the captive bubble method of both membranes. As the measurements were conducted underwater, any effects of swelling or evaporation were eliminated. Thus, representing more realistic membrane conditions. For a drop base diameter of 0.8 mm, the resulting contact angles were 20° for the M1 membrane and 15° for M2, respectively, while for a bubble volume of 0.2 mm, the measured angles were 32° and 28°, respectively. There was no observable inflexion to account for, as the smaller bubble did not induce significant stretch. These observations agreed with other reported studies and the hypothesis that the size dependence of the contact angle occurs only on rough and heterogeneous surfaces^43^. The contact angle of the M1 membrane reduced rapidly in the first 20 s (Fig. 3). At M2, the contact angle decreased slowly while the droplet base remained constant. The time dependency indicated the rates of surface spreading and absorption or capillary imbibition into the porous structure of the sponge.

#### Low-temperature nitrogen adsorption-desorption isotherm

The porosity and specific surface area of the newly developed membrane were measured using nitrogen adsorption-desorption isotherms at 77 K (Fig. 3) using Brunauer, Emmett and Teller (BET) equation. The results indicated that both membranes were microporous materials, with type IV isotherms observed. Compared with M1, the M2 membrane showed a more gradual adsorption slope and a much smaller hysteresis loop, indicating finer and more homogeneous pores. The BET specific surface area, total pore volume, and mean pore diameter of the membranes are summarised in Supplementary Table S1. Average pore diameters determined by BET were in the nanometer range (≈ 2 nm), much smaller than the micrometer-scale pores seen by SEM (Fig. 1C). The structure also displayed narrow hysteresis loops (Fig. 3a), indicating a highly ordered and well-defined structure^44^. Pore distribution analysis confirmed the presence of mesopores (Fig. 3b), while the pore distribution in the M2 membrane revealed the existence of different pores throughout the entire membrane surface (Fig. 3b). M2 had a higher specific surface area, indicating more accessible pores for enhanced adsorption capacity and surface interactions. On the other hand, M1 had a significantly larger total pore volume, providing more space for high storage or flow-through capacity. Despite having a higher specific surface area, M2 had a much lower total pore volume, indicating a denser structure with more surface interaction sites. This behavior can be attributed to the presence of PEG and PVP additives, which promoted phase separation during membrane formation and increased water uptake in the polymer-rich regions, resulting in a sponge-like structure with controlled pore formation. Moreover, the desorption branch suggested that the pores may be interconnected, contributing to capillary effects. These findings suggest that the M2 membrane may be more suitable for applications where surface interactions are critical, despite its lower overall pore volume.

It should be noted that SEM only showed the macroscopic surface features of the polymer layer, while BET analysed the overall surface area and pore structure of the entire membrane, including the support. This highlighted the difference between observed macropores (Fig. 1C) and nanoscopic pores (Fig. 3). Based on these analyses, the modified membrane (M2) exhibited a more uniform and stable pore structure with a broader size distribution, making it better suited for filtration experiments and applications that required higher permeability and flux than M1.

### Permeability–selectivity characterisation of PMMA/pPFPA/PEG/PVP/TiO_2, mod_ membrane

The objectives of this study are to investigate the rejection of Pharma Mix through (i) cross-flow setup, (ii) sorption setup, and (iii) photocatalysis setup.

**Fig. 4:**
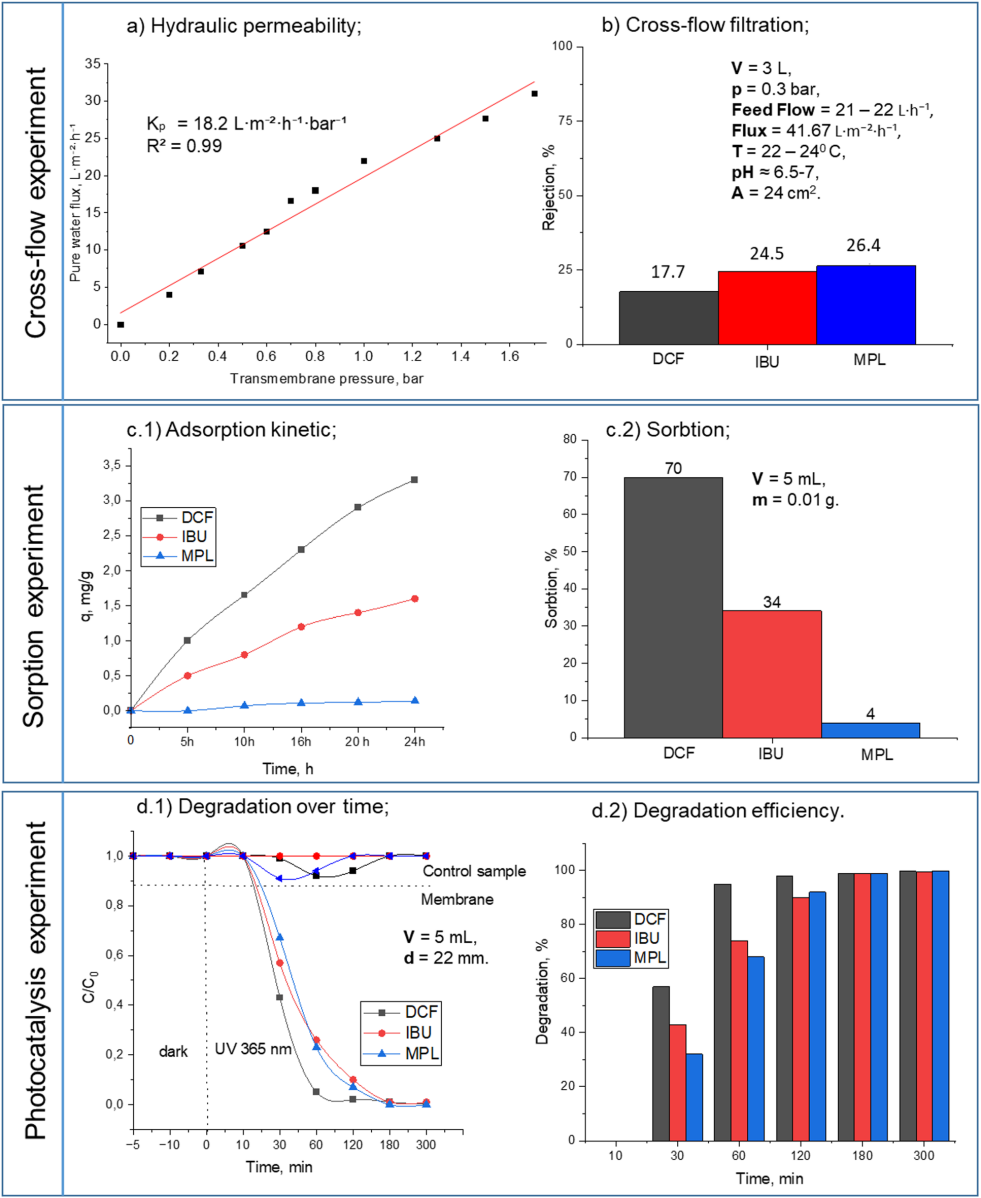
Separation experiments with PMMA/pPFPA/PEG/PVP/ TiO_2, mod_ membrane (concentration of Pharma Mix was DCF – 1 mg L^− 1^, IBU – 1 mg L^− 1^, MPL – 1 mg L^− 1^).

During a cross-flow experiment with two membranes, it was discovered that the surface of PMMA/pPFPA/TiO_2−mod_ membrane was not stable and was completely washed off the support immediately after the start, making it impossible to conduct the experiment. The flux values increased linearly with pressure but remained lower compared to M2 due to the lower maximum permeability. M2 showed a higher flux range due to its higher surface area, hydrophilicity and permeability, making it more efficient under the same pressure conditions. Thus, further separation experiments with Pharma Mix will be conducted only for PMMA/pPFPA/PEG/PVP/TiO_2, mod_ membrane. Figure 4 summarises the results of the separation experiments.

#### Hydraulic permeability

The linear relationship between permeate flux and transmembrane pressure was maintained up to approximately 1.8 bar. Therefore, the hydraulic permeability coefficient (K_p_) was calculated (Eq. 4) from the slope of the linear region only, excluding the highest-pressure point where deviation from linearity was observed. A strong linear correlation was observed (R² = 0.98), yielding K_p_ = 18.2 L·m^− 2^·h^− 1^·bar ^− 1^. The obtained values are displayed in Fig. 4a.

The moderate K_p_ is consistent with the measured porosity and low total pore volume (0.0023 cm^3^·g^− 1^). According to the Hagen–Poiseuille/Darcy framework, hydraulic permeability scales proportionally with ε·r² (where ε is porosity and r is effective pore radius). Despite the small pore size, the membrane exhibited notable water permeability, indicating that water transport is not governed solely by the micropores detected by BET analysis, but is also influenced by the interconnected pore network observed in SEM images (0.30 ± 0.11 μm). It is worth mentioning that not only morphology but also other factors, such as hydrophilicity, have an effect on the hydraulic permeability of membranes^45^. The hydrophilic contact angle (28°) and negative surface charge (− 31 mV) increased the water uptake capacity of the membrane, contributing to the linear Darcy regime observed up to 1.8 bar and supporting stable hydraulic performance. The flux variations at the outlet are in accordance with the applied membrane pressures. As shown in the diagram, the permeate flow increased proportionally with increasing transmembrane pressure within the linear regime. The higher permeability of the membrane can be attributed to its interconnected porous structure and low contact angle, rather than pore size alone.

#### Crossflow filtration experiments

The TiO_2, mod_. content was up to 0.5 wt% based on Fig. 4b), the pore size was too large to reject pharmaceuticals effectively. This led to a high number of DCF, IBU, and MPL molecules passing through the pores, resulting in a decreased membrane retention rate. The cross-flow hydrodynamic conditions affect the selective deposition of smaller particles or colloids, which are more likely to be deposited on the membrane. Due to the balance between convection flow and particle backscattering, larger molecules (like DCF 296 g mol^− 1^) with higher backscattering speeds tend to move away from the membrane surface, while smaller particles (like MPL 206 g mol^− 1^) are more likely to be deposited as soiling agents. The removal efficiency by the membranes can be ranked as MPL > IBU > DCF. However, during crossflow experiments, the sorption was reduced, which could contribute to the membranes’ antifouling properties, leading to an increased retention rate.

#### Sorption

The 24-h sorption experiment of Pharma Mix had shown good sorption on the membrane surface. Negatively charged DCF—70% and neutral IBU—50%. This result is fully connected with the point zero charge of the membrane surface. Moreover, the membrane’s hydrophilicity could prove the high number of adsorbed pharmaceutical molecules (Fig. 4c.1-c.2).

#### Photocatalysis

The membrane surface had reliable photocatalytic properties. We determined the photocatalytic degradation after 30 min of pre-adsorption in the dark (Fig. 4d. 1). No noticeable degradation occurred without light, indicating the need for external light to initiate degradation. After three hours of irradiation, we observed complete photocatalytic degradation of all pharmaceuticals in the mixture. TiO_2_ has a wide band gap energy of 3.0–3.2 eV and can only be excited by UV light with a wavelength of less than 387 nm^46^. When the polymer composition is incorporated with TiO_2, mod_. nanoparticles, it increases light absorption and modifies the electron density^47^. As a result, the membrane can provide a large excitation binding energy and absorb over a large region of the UV spectrum. The 100% degradation indicated that the surface contained active photocatalytic nanoparticles that were not fully covered by the polymer. To investigate the impact of incorporating TiO_2, mod_. into the photocatalytic membrane matrix, we also tested a membrane without TiO_2, mod.,_ under the same conditions. Figure 4. d1) shows the change in Pharma Mix concentration with UV irradiation time in the presence of membranes. The results demonstrated that membranes with TiO_2, mod_. showed significantly higher photocatalytic activity compared to the pristine support. Photodegradation of pharmaceuticals increased after 30 min, with degradation rates following the sequence DCF > IBU > MPL, reaching 100% decomposition rates after 300 min (Fig. 4d. 2). The curves depicting reduced concentration displayed an exponential pattern, indicating that the degradation kinetics of the pharmaceuticals follow a pseudo-first-order scheme^48^. Pharmaceuticals were presented in low initial concentrations (C_0_), so the degradation adheres to the Langmuir–Hinshelwood (L–H) law^49^.

TiO_2_ release remained minimal under all tested conditions, including static sorption, photocatalysis and hydraulic cross-flow. The highest detected concentration (0.012 mg·L^− 1^) corresponded to < 0.05% of the total TiO_2_, confirming strong nanoparticle immobilisation (Supplementary Table S2).

The separation results for PMMA/pPFPA/PEG/PVP/TiO_2, mod_ membrane showed that the removal efficiency of pharmaceuticals was significantly lower in the cross-flow experiment compared to sorption and photocatalytic degradation. It’s important to note that a direct comparison between these experimental setups may not be entirely fair due to differences in membrane area and pharmaceutical solution volume. In the cross-flow experiment, the solution circulated continuously between the flask and the tubes. The membrane area and the volume of the Pharma Mix increased from the sorption and photocatalyst experiments to the cross-flow experiment (from 4 cm^2^ to 22 cm^2^ and from 5 mL to 3 L).

**Fig. 5:**
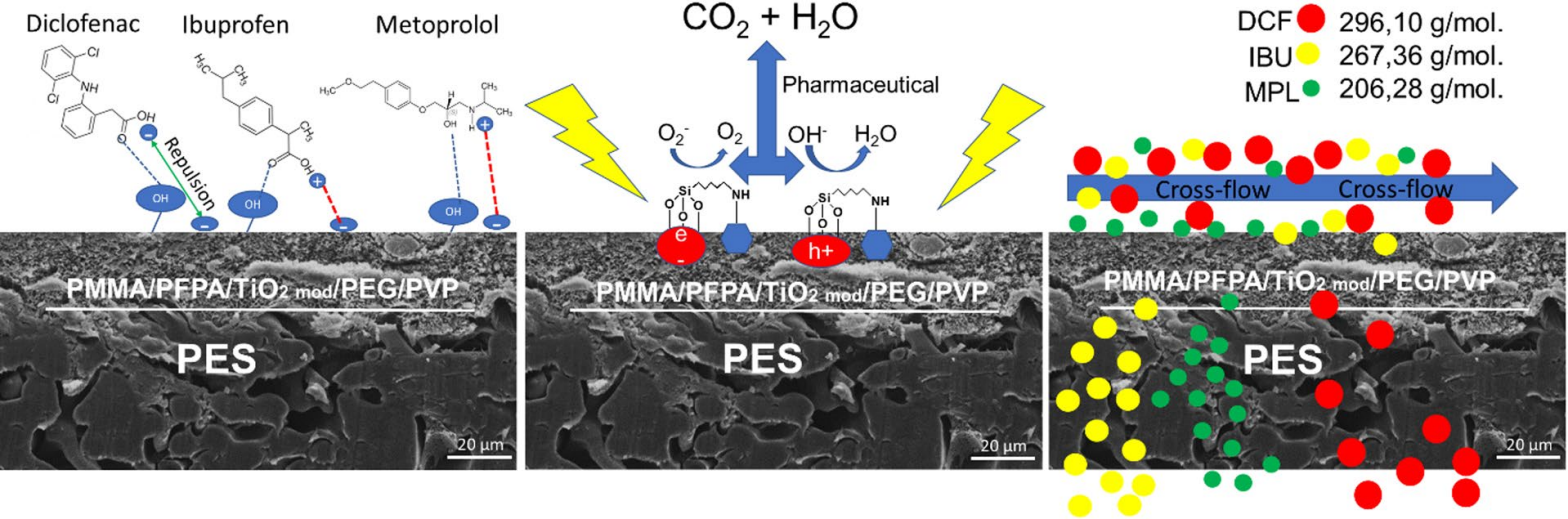
Proposed mechanism of Pharma Mix retention using PMMA/pPFPA/TiO_2, mod_. /PEG/PVP membrane. (**a**) Sorption behavior, (**b**) photocatalytic degradation, (**c**) cross-flow and sieving effect.

Considering different separation methods, it could be seen that the results with the same membrane distinguished different pharmaceuticals (Supplementary Table S.3). A proposed mechanism of membrane separation experiments is presented in Fig. 5a–c).

A rapid initial adsorption (Fig. 5a) occurs due to many active sites on the membrane surface. Adding PEG to the polymer composition increases the number of hydroxyl groups on the membrane surface. While PEG primarily acts as a porogen and was partially washed-out during membrane formation, at the high content used here (25 wt%), residual PEG can contribute to the overall –OH signal (Fig. 1B). These hydroxyl groups, along with those from TiO_2 mod_, and the formed carboxylic groups by hydrolyzed pPFPA can interact with pharmaceutical molecules in water mainly through hydrogen bonding and hydrophobic interactions, rather than acting as electron acceptors. The surface of the TiO_2, mod_ was amphoteric, which means that its adsorption is influenced by pH. However, FTIR (Fig. 1B) and IEP (Fig. 2B) confirmed that under the studied conditions, the overall surface charge of the membrane is negative. Since both the membrane surface and DCF are negatively charged, electrostatic repulsion dominates; therefore, DCF adsorption occurs mainly through non-electrostatic mechanisms such as hydrogen bonding, hydrophobic, or π–π interactions. As for IBU, it is also negatively charged at pH 7 (pKa ≈ 4.9), so its adsorption is primarily due to interactions with the OH groups on the membrane surface. Nanoparticles contribute to higher IBU adsorption because their smaller size facilitates adsorption^44,50^. The TiO_2, mod_. nanoparticles on the membrane surface repel MPL molecules due to their positive charge, leading to lower adsorption. It’s worth mentioning that the different sizes of the three pharmaceutical molecules affect their sorption on the membrane surface. The largest molecule, DCF, forms a cake layer on the membrane surface, making it difficult for smaller molecules to be adsorbed. These sorption results differ from the findings of other researchers, Childress et al.^51^, Elimelech et al.^52^, and Boussu et al.^53^, where cationic pollutants led to the formation of a cake layer. In our study, we support the findings of Sagle et al.^54^, which show that negatively charged surfaces have less sorption with negatively charged pharmaceuticals, while less negatively charged surfaces have more sorption with negatively charged pharmaceuticals under given conditions. Indeed, PMMA/pPFPA/PEG/PVP/TiO_2, mod_ membrane sorption properties agreed with PZC and followed the order MPL < IBU < DCF.

Figure 5b presented the proposed mechanism of photocatalytic degradation of pharmaceuticals on the membrane surface. The TiO_2, mod_ nanoparticles on the membrane interface, created holes (h^+^ _vb_) in the valence band and electrons (e^−^ _cb_) in the conduction band upon irradiation. The process is slower in acidic conditions than in neutral conditions. We suppose that under neutral conditions, the membrane will produce the hydroxyl radical (OH•) according to the:

$$\:{\mathrm{h}}_{\mathrm{v}\mathrm{b}}^{+}+{\mathrm{H}}_{2}\mathrm{O}\to\:\:{\mathrm{O}\mathrm{H}}^{{\cdot\:}}+{\mathrm{H}}^{+}$$

DCF, IBU, and MPL levels decreased rapidly and almost completely disappeared after a 2-h reaction.

We believe that the degradation of DCF occurred through the generation of hydroxyl derivatives, ring opening, and eventual mineralization^55^. In Fig. 4d2), it was observed that IBU was almost completely degraded in the presence of photocatalysts after 3 h of UV light exposure. Skoumal et al.^56^ noted that the presence of a free radical (OH•) could lead to the formation of monohydroxylated IBU, which could then be oxidised to produce carbonyl compounds (aldehydes and ketones)^57^. The MPL photocatalytic degradation on the membrane surface may be due to oxidation with ring cleavage to produce low-molecular-weight organic acids before completely mineralising to CO_2_ and water^58^. This is proof of the existence of OH• radicals on the membrane surface, and it is considered that OH• contributes 100% to degradation^59^.

Figure 5c displayed the separation mechanism during cross-flow filtration, where pharmaceuticals are fractionated by selective deposition^60^. The relative magnitude of average pore size diameter, size difference of transferable and nontransferable molecules, and pore network structure determined the main filtration mechanism for a membrane process. According to BET analysis, the presence of micropores in the PMMA/pPFPA/PEG/PVP/TiO_2, mod_. membrane (2 nm) results in convective flux. MPL has a lower molecular weight because cross-flow hydrodynamic conditions influence the selective deposition of MPL to the membranes. In such a case, the membrane can serve as a barrier to separate pharmaceuticals in a mixture. Due to the balance between the convection flow and the backscattering, higher molecular weight molecules like DCF and IBU, which are characterised by higher backscattering speeds, tend to move away from the surface of the membrane. In contrast, the lower molecular weight MPL was preferably deposited.

In summary, our membrane demonstrates high photocatalytic efficiency with minimal TiO_2_ loading, achieving 100% degradation of pharmaceuticals within 2 h under UV irradiation. In contrast, a conventional TiO_2_/PVDF mixed-matrix membrane^15^ degraded DCF by only ~ 55% after 120 min, while ibuprofen showed negligible removal, and TiO_2_PVDF–P25 membranes^14^ showed metoprolol removal of 35–38%.

## Conclusion

PMMA/pPFPA/PEG/PVP/ TiO_2, mod_ membrane was produced using high-molecular-weight additives, such as PEG and PVP, which formed a more uniform, sponge-like pore structure. In this work, we showed the combination of two systems in a single membrane platform. Hydrophilic moieties like PEG and PVP were incorporated into hydrophobic membrane to tailor porosity and water flux, while TiO_2 mod_ provided photocatalytic degradation of drugs. With this approach, we address the drawbacks of both systems by achieving covalent and physical crosslinking of the hydrophilic segments and TiO_2, mod_, which improves stability and performance at low nanoparticle loadings.

The interactions between PVA and PEG were confirmed by FTIR bands as well as the SEM images of the structure. BET analysis also established a more uniform pore structure.

The current membrane was used for separation experiments against the pharmaceutical mixture (DCF, IBU, and MPL). The cross-flow rejection rate generally exceeded 30% for all the pharmaceuticals. The adsorption capacity of DCF reached the highest value, followed by IBU and MPL. Meanwhile, during photocatalysis, degradation of all three pharmaceuticals in the Pharma Mix reached almost 100% after two hours of UV exposure. Contaminant removal in this system relies mainly on adsorption and photocatalytic degradation, highlighting its suitability for low-pressure reactive filtration and photocatalytic membrane use.

The study represents a significant contribution to the field of membrane production, as the developed PMMA/pPFPA/PEG/PVP/TiO_2, mod_ membrane could be used as a high-performance photocatalytic membrane. In our upcoming experiments, we need to adjust the UV irradiation for the cross-flow cell. It is crucial to optimise this setup to achieve higher degradation rates.

## Materials and methods

### Materials

Pentafluorophenol (Apollo Scientific), triethylamine (> 99%, Fisher Scientific), 3-mercaptopropionic acid (99%, Thermo Scientific), acetone (99%, Fisher Scientific). acryloyl chloride (stabilised with phenothiazine, 96%, Merck), carbon disulfide (99.9%, Merck), dichloromethane (> 99.5%, Roth), methanol (99%, Merck), potassium phosphate anhydrous (97%, Sigma-Aldrich), 2-bromoisobutyric acid (98%, Sigma-Aldrich). TiO_2_ nanoparticles (anatase, 25 nm), 3-aminopropyltriethoxysilane (APTES, 99%, Roth), polyethylenoxid (F.W.0.0, M.W. 200 000), polyvinylpyrrolidone (M.W. 50 000) and poly(methyl methacrylate) (M.W. 350 000) was purchased from Sigma Aldrich. DMF 99.8%, Acros Organics).

The pharmaceuticals used were Metoprolol (MPL), Ibuprofen (IBU), and Diclofenac (DCF). Their main characteristics are presented in Supplementary Table S4.

### Sythesis of poly(pentafluorphenylacrylate)

Pentafluorophenylacrylate was synthesised as described^61^. The iniferter RAFT initiator 2-((((2-carboxyethyl)thio)carbonothioyl)thio)-2-methylpropanoic acid was synthesised as described^62^. 2.38 g (10 mmol, 1 eq.) of as prepared PFPA was dissolved in 1 mL of DMF. Subsequently, 25 µL (0.000186 mmol, 0.0000186 eq.) of a 1 mg/mL iniferter RAFT initiator stock solution was added. The reaction solution was purged with nitrogen for 15 min under cooling with an ice bath. The polymerization was carried out at ambient temperature for 7 h in a home build photoreactor with 2 × 4 W 365 nm LEDs. The polymer was recovered as a white powder by precipitation in MeOH several times (560 mg).

### Fabrication of pPFPA/PMMA membrane with modified TiO_2_ nanoparticles

TiO_2_ nanoparticles were modified using APTES according to the method described^63^. The dope solution was prepared by mixing appropriate amounts of pPFPA, PMMA and TiO_2, mod_ in DMF at 70 °C, continuously stirred for 3 h until a homogeneous solution was obtained (Table 1).

The solution was then cast onto a THF-conditioned polyester nonwoven support (type FLPD 85, Freudenberg Performance Materials) on a glass plate using a doctor blade with a 200 μm gap. The nascent membrane was immersed in the coagulation bath (a mixture of water and ethanol 30/70 vol %) for 15 min and then dried at 40 °C. After coagulation, the membrane was transferred into freshly collected DI water and kept overnight to remove any remaining solvent.

The PMMA/pPFPA/TiO₂ mod. (M1) membrane was prepared from this hybrid polymer solution. Fine-tuning of the pore structure was achieved by adding high-molecular-weight additives (PEG and PVP), resulting in the formation of a PMMA/pPFPA/TiO₂ mod./PEG/PVP (M2) membrane. The membrane compositions are shown in Table 1.

**Table 1:** Composition of the solutions used for membrane fabrication.

Membrane	Polymers, wt%		Additives, wt%			Solvent, wt%
	PMMA	pPFPA	PEG	PVP	TiO_2 mod_.	
M1	6	6	0	0	0.5	87.5
M2	6	6	25	5	0.5	57.5

### Membrane morphology characterisation

*Fourier-transform infrared spectroscopy (FTIR)* was conducted using a PerkinElmer FTIR spectrophotometer (USA) in the attenuated total reflectance (ATR) mode. Spectra were recorded over the range of 4000–400 cm⁻¹ with a resolution of 4 cm⁻¹, averaging 32 scans per sample to improve the signal-to-noise ratio. All measurements were performed at room temperature, and the samples were carefully dried prior to analysis to avoid interference from adsorbed moisture.

*Scanning electron microscopy (SEM)* were analysed using VEGA 3 device; TESCAN GmbH, Dortmund, Germany. The samples were mounted on an aluminium stud using adhesive carbon tape and were sputter-coated with approximately 6 nm platinum using a BAL-TEC MED 020 sputter coater connected to a BALTEC MCS 010 and a BAL-TEC QSG 060 (BAL-TEC AG, Pfäffikon, Switzerland).

*Low-temperature nitrogen adsorption–desorption isotherms* were used for the analysis of the specific surface area, pore volume, and pore-size distribution of the newly developed membrane. Isothermal adsorption and desorption of Nitrogen on the surfaces of these materials were performed. NOVAtouch Gas Sorption System Model 1–4, provided by Anton Paar QuantaTec, Inc., Florida, USA, was used. Software TouchWin™ Version 1.22 [Build 18] (available at: https://www.anton-paar.com) was used to interpret the data. Adsorption isotherms for nitrogen at 77 K were performed considering the following physical constants: Molecular Weight 28.013 g∙mol^− 1^; cross-section area 16.2 Å^2^∙mol^− 1^, non-ideality 6.58 × 10^− 5^ 1/torr. A cell type with an outer diameter of 9 mm was used for all measurements. 40 points for each adsorption and desorption curve were recorded over the whole relative pressure range P/P_0_ between 0 and 1. Helium was used as a medium for the measurement of the Void Volume Mode.

### Membrane surface charge characterisation

*Contact angle measurements using the captive bubble method* were used to determine membrane surface hydrophobicity with the OCA 25 device (DataPhysics Instruments GmbH). The contact angle of the membrane was evaluated using the captive bubble method^64^. The contact angle could be evaluated by a simple equation: 180° − below and of the inflexion angle above, independent of the bubble volume.

*The zeta potential of membranes and the membrane’s surface charge* were determined using Zeta Potential Analyzer ZPA 20 (DataPhysics Instruments) . A KCl electrolyte solution was used by dissolving 0.015 g of KCl in 250 ml of DI water. The pH was adjusted using 0.1 M of NaOH and HCl to measure the streaming potential.

### Permeability–selectivity characterisation

#### Separation experiments of PMMA/pPFPA/PEG/PVP/TiO₂ modified membranes

*Hydraulic Permeability* were carried out using a self-made cross-flow membrane cell with an effective filtration area of 22 cm^2^. Under steady state, the permeated flux was recorded over time at different operating pressures ranging from 0.2 to 2 bar.

*Pure water permeability (Pw)* was calculated with the following equation (Eq. 1):

1 $$\:{P}_{w}=\frac{Q}{A\varDelta\:t\varDelta\:P}$$

where Q is the volume of permeate water (L), Δt is the collection time (h), A is the effective membrane area for filtration (m^2^), and ΔP is the transmembrane pressure (bar). All the Pw (L·m⁻²·h⁻¹·bar⁻¹) is calculated at different pressures, and the reported data represents an average value.

*Water flux (J)* was calculated according to Eq. 2:

2 $$\:J=\frac{V}{A\cdot\:t}$$

where V is the volume of permeate collected (L), A is the effective membrane area (m²), and t is the filtration time (h). The resulting flux is expressed in L·m⁻²·h⁻¹.

*The hydraulic permeability* of the membranes was determined according to Darcy’s law, Eq. 3:

3 $$\:J={K}_{p}\cdot\:{\Delta\:}P$$

where *J* is the permeate flux (L·m^− 2^·h^− 1^), *K*_*p*_ is the hydraulic permeability coefficient (L·m^− 2^·h^− 1^·bar ^− 1^), and ΔP is the transmembrane pressure (bar).

The hydraulic permeability coefficient (*K*_*p*_) was experimentally determined as the slope of the linear regression of pure water permeate flux (*Jw*) versus transmembrane pressure (TMP).

*Crossflow filtration experiments* were performed using semi-technical scaled membrane units, such as test cells and 4”-modules, and necessary equipment for membrane operation (Supplementary Fig. S1). The filtration cell is realised with two parallel 3 cm thick glass plates with a length of 21 cm and a width of 15.5 cm. The membrane is pressed parallel to the plates and secured across the full width of the flow channel. The size of the membrane cell is 4 × 12 cm. Fluid is injected through 2 tubes located at the top of the plate. The setup is fully saturated with feed solution (FS) in the initial phase. During the experiment, FS circulated between plates. Flow rate and pressure can be designed during the experiment. Samples are taken from the FS and the filtrate. Cross-flow filtration experiments were conducted for 2 h.

The pharmaceutical mixture (Pharma Mix) consists of DCF 1 mg/L, MPL 1 mg/L, and IBU 1 mg/L. Samples were taken from the feed water and filtered (Fig. S2). Operation conditions are 0.33 bar pressure, feed flow 21–22 L/h, flux 41,6 L·m⁻²·h⁻¹, temperature 22–24 °C., pH ≈ 7.

The following Eq. 4 calculated the retention rate (R) of the Pharma Mix:

4 $$\:R=\left(1\mathrm{-}\frac{{C}_{p}}{{C}_{f}}\right)\cdot\:100$$

where $\:{C}_{p}$ and $\:{C}_{f}$ are the Pharma Mix concentrations in permeate and feed, respectively.

*Sorption experiments* were carried out with 0.01 g of the membrane added to 5 ml of Pharma Mix at pH ∼6.0 in a dynamic mode, with constant shaking to ensure proper mixing. Samples were collected after 24 h.

*Photocatalysis experiments* with Pharma Mix were used to evaluate the membrane’s photocatalytic properties. The membranes (20 mm in diameter) were completely submerged in the pollutant solutions (5 mL, pH 6.5) and left in a dark condition for 1 h to reach the adsorption equilibrium. The photocatalytic degradation was initiated using UV irradiation (100 W, 365 nm, LED-Light, China) for 3 h while the pollutant solutions were in static mode. The distance between the UV source and the membrane surface was 10 cm. Aliquots were taken at regular time intervals (30 min). The rate constant for the photodegradation reaction was determined by dividing the concentration ($\:{C}_{0}$) at the initial time by the concentration ($\:{C}_{t}$ ) at reaction time t, according to Eq. 5:

5 $$\:k=\frac{{C}_{0}}{{C}_{t}}$$

*High-performance liquid chromatography (HPLC)* was used to measure concentrations in feed and permeate solutions on a Dionex UltiMate 3000 HPLC system (Thermo Fisher Scientific) equipped with an EC 150/3 Nucleodur C18 PAH, 3 μm (Macherey-Nagel) column. The mobile phase flow rate was 0.35 mL/min, with a total run of 20 min. The detection wavelength for chemical impurities was 196–220 nm. Retention time: Metoprolol − 2,16 min.; Diclofenac − 13,20 min.; Ibuprofen − 15,69 min.

*TiO*_*2*_ *leaching analysis* was evaluated using UV–Vis spectrophotometry (PerkinElmer Lambda 35, USA). TiO_2_ exhibits a characteristic absorption band in the range of approximately 350–370 nm. The absorbance of the collected permeate at this wavelength was measured and calibrated against standard TiO_2_ suspensions of known concentration to estimate nanoparticle release.

## Supplementary Information

Below is the link to the electronic supplementary material.

Supplementary Material 1

## Data Availability

All data generated or analysed during this study are included in this published article (and its Supplementary Information files).
