# Supplementary material for: PMMA/pPFPA membrane with low content of modified TiO2 nanoparticles for effective retention of pharmaceuticals from water
Source: Sci Rep. 2026 Mar 26;16:10506. doi: 10.1038/s41598-026-45387-3 (PMC13031542; doi:10.1038/s41598-026-45387-3)
Supplement: Supplementary file 1 — Supplementary Material 1 [file 41598_2026_45387_MOESM1_ESM.docx]

**PMMA/pPFPA membrane with low content of modified TiO_2_ nanoparticles for effective retention of pharmaceuticals from water**

**Mariia Pasichnyk^1,2,*^, Christian Schmitt^3^, Martina Plank^3^, Kristin Kerst^1,*^, Wei Wang^4^, Muhannad Al Aiti^4^, Gianaurelio Cuniberti^4^, André Lerch^1^ and Patrick Théato*^2,3^**

^1^Chair of Process Engineering in Hydro Systems, TUD Dresden University of Technology, Dresden, 01069, Germany

^2^ Institute for Technical Chemistry and Polymer Chemistry (ITCP), Karlsruhe Institute of Technology (KIT), Engesserstraße 18, Karlsruhe, 76131, Germany

^3^ Institute for Biological Interfaces III (IBG-3), Soft Matter Synthesis Laboratory, Karlsruhe Institute of Technology (KIT), Hermann-von-Helmholtz-Platz 1, Eggenstein-Leopoldshafen, 76344, Germany

^4^ Chair of Material Science and Nanotechnology, TUD Dresden University of Technology, Dresden, 01062, Germany

*corresponding author: [mariia.pasichnyk@tu-dresden.de](mailto:mariia.pasichnyk@tu-dresden.de)

*corresponding author: [kristin.kerst@tu-dresden.de](mailto:kristin.kerst@tu-dresden.de)

*corresponding author: [patrick.theato@kit.edu](mailto:patrick.theato@kit.edu)

**Table S1.** BET analysis of membrane M1 and M2.

| **Name** | **Specific surface Area m²∙g^-1^** | **Total Volume cm³∙g^-1^** | **Mean Pore Diameter nm** |
| --- | --- | --- | --- |
| M 1 | 57.99 | 0.2278 | 1.68 |
| M 2 | 71.07 | 0.0023 | 1.70 |

**Table S2.** TiO_2_ leaching under different operating conditions

| **Operating Mode** | **UV Irradiation** | **Flow Condition** | **Time**  **(h)** | **Absorbance (360 nm)** | **TiO_2_ Concentration (mg/L)** | **Total TiO_2_**  **(%)** |
| --- | --- | --- | --- | --- | --- | --- |
| Sorption | No | Static | 24 | 0.0018 | 0.008 | <0.03 |
| Photocatalysis | Yes | Static | 3 | 0.0023 | 0.011 | <0.05 |
| Cross-flow | No | Cross-flow | 2 | 0.0020 | 0.009 | <0.04 |

**Table S3.** Removal performance and dominant mechanisms for selected pharmaceuticals using the photocatalytic membrane.

| **Pharmaceutical** | **Molecular Weight (g/mol)** | **Cross-flow Rejection**  **(%)** | **Adsorption / Sorption**  **(%)** | **Photocatalytic Degradation (%)** | **Dominant Removal Mechanism** |
| --- | --- | --- | --- | --- | --- |
| Diclofenac | 296.10 | 17.7 ± 0.3 | 70 ± 0.2 | ~100 | Photocatalysis / Sorption |
| Ibuprofen | 267.36 | 24.5 ± 0.5 | 34 ± 0.3 | ~100 | Photocatalysis / Sorption |
| Metoprolol | 206.28 | 26.4 ± 0.2 | 4 ± 0.2 | ~100 | Photocatalysis / minimal size-exclusion |

**Table S4.** Pharmaceuticals that were used.

| **Name** | **Formula** | **Molecular weight,**  ***(*g/mol*)*** | **Solubility in water, 25^0^ C**  ***(*mg/L*)*** | **pKa** | **Log**  **Kow** | **Surface Area,**  **(Å²)** |
| --- | --- | --- | --- | --- | --- | --- |
| Metoprolol (MPL) | C_34_H_56_N_2_O_12_ | 267.36 | >1000 | 9.56 | 1,88 | 50.7 |
| Ibuprofen (IBU) | C_13_H_18_O_2_ | 206,28 | 21 | 5.2 | 3,97 | 37.3 |
| Diclofenac (DCF) | C_14_H_11_Cl_2_NO_2_ | 296,1 | 2.37 | 4.2 | 4,51 | 49.3 |


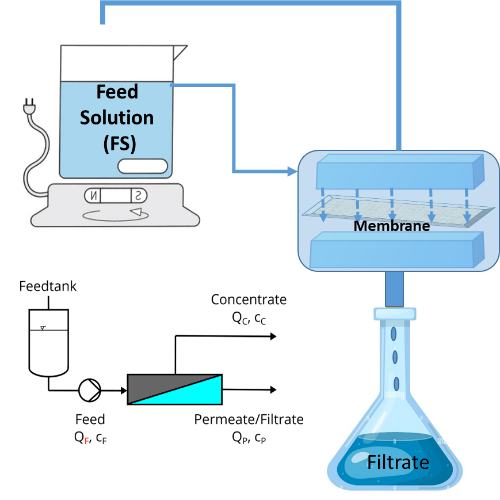


**Figure S1.** Cross-flow filtration setup used for water flux measurement of the membrane and for separation of the pharmaceutical mix.
